# Blood-based biomarkers of frailty in solid tumors: a systematic review

**DOI:** 10.3389/fpubh.2023.1171243

**Published:** 2023-05-04

**Authors:** Dilorom Sass, Brennan Parmelee Streck, Vivian A. Guedes, Diane Cooper, Jennifer L. Guida, Terri S. Armstrong

**Affiliations:** ^1^Neuro-Oncology Branch, Center for Cancer Research, National Cancer Institute, National Institutes of Health, Bethesda, MD, United States; ^2^Basic Biobehavioral and Psychological Sciences Branch, Behavioral Research Program, Division of Cancer Control and Population Sciences, National Cancer Institute, National Institutes of Health, Rockville, MD, United States; ^3^Office of Research Services, National Institutes of Health Library, National Institutes of Health, Bethesda, MD, United States

**Keywords:** biomarkers, molecular biomarkers, solid tumors, frailty, deficit accumulation, cancer survivors

## Abstract

This review examines the current literature to identify biomarkers of frailty across patients with solid tumors. We conducted the systematic review using preferred reporting items for systematic reviews and meta-analysis guidelines (PRISMA). PubMed, Web of Science, and Embase databases were searched from their inception to December 08, 2021, for reports of biomarkers and frailty. Two reviewers independently screened titles, abstracts, and full-text articles. A quality assessment was conducted using NHLBI Quality Assessment Tool for Observational Cohort and Cross-Sectional Studies, and Quality Assessment of Case-Control Studies. In total, 915 reports were screened, and 14 full-text articles were included in the review. Most studies included breast tumors, were cross-sectional in design, and measured biomarkers at baseline or pre-treatment. Frailty tools varied with Fried Frailty Phenotype and the geriatric assessment most frequently used. Increased inflammatory parameters (i.e., Interleukin-6, Neutrophil Lymphocyte Ratio, Glasgow Prognostic Score-2) were associated with frailty severity. Only six studies were rated as good quality using assessment ratings. Together, the small number of studies and heterogeneity in frailty assessment limited our ability to draw conclusions from the extant literature. Future research is needed to identify potential target biomarkers of frailty in cancer survivors that may aid in early detection and referral.

## 1. Introduction

Cancer and cancer therapies may contribute to the development of early onset frailty, a geriatric syndrome that is indicative of multi-system decline and often precipitates mortality (1–4). The prevalence of frailty has been reported to range from 8% in adult survivors of childhood cancer to 59 percent in older adult cancer survivors using phenotypic and deficit accumulation frailty measures (5). Sustained or worsening phenotypic frailty measured prior-to post-cancer diagnosis significantly increases the risk of mortality in patients with solid tumors (breast, lung, colorectal, ovarian, and endometrial) (6). Thus, there is an increased need for early identification of patients at risk for developing frailty to aid in timely therapeutic interventions.

Two commonly used, but conceptually distinct constructs of frailty, include: (i) phenotypic frailty, where frailty is a defined and measurable state (e.g., fried frailty phenotype) (7) and (ii) the accumulation of deficits, where frailty is more of a stochastic process, in which random deficits lead to increased vulnerability (8). While phenotypic frailty evaluates signs/symptoms (e.g., weight loss, exhaustion, and weakness) and may exist independent of medically classified conditions as a pre-disability syndrome, deficit accumulation frailty is based on a long checklist of signs/symptoms and medically classified conditions, including disability (9). Phenotypic frailty is most useful if the goal is to define risk factors and mechanisms with a degree of specificity for sub-clinical and clinical frailty because individuals are stratified into distinct risk categories and specific pathways can be identified for prevention and remediation. Stochastic deficit accumulation frailty may be helpful for individual prognostication and targeting shared risk factors or biological mechanisms (10). To encompass the two conceptual definitions, in this review, frailty will be operationalized as both phenotypic frailty (7) and deficit accumulation frailty (8).

Cancer and cancer treatments may accelerate aging which may be measured using correlates or biomarkers representative of hallmarks of aging (e.g., telomere attrition, epigenetic alteration, loss of proteostasis, deregulated nutrient sensing, mitochondrial dysfunction, senescence, and inflammation) and may in turn lead to early frailty states (2, 5, 11). Indeed, several studies report that completion of primary cancer therapy (post-treatment) accelerates biological aging in cancer survivors, as evidenced by increased expression of cytokines (12, 13), senescence-associated p16^INK4^ (13), and decreased telomere length (14). However, little is known about the association of these biological measures of aging with frailty in cancer survivors with solid tumors. For example, several recent reviews and/or meta-analyses evaluated common frailty biomarkers in older adults, but few included oncologic studies (15–18). The search for sensitive and specific biomarkers of frailty in oncological populations is crucial for early detection of aging-related consequences of cancer and its treatments on cancer survivors (2). Such biomarkers may offer diagnostic and prognostic utility by aiding clinical assessment of frailty signs/symptoms and may help evaluate the effectiveness of interventions designed to mitigate (or potentially reverse) phenotypic and deficit accumulation frailty. Given the heterogeneity in the biology, treatments, and frailty rates (19, 20) between hematologic and solid cancers, this review evaluates biomarkers of frailty specific to cancer survivors with solid tumors.

Potential target biomarkers of frailty may be used to identify cancer survivors at risk for the development of frailty. To fill this gap, this systematic review synthesizes current literature by examining (i) frailty measures and (ii) biomarkers evaluated in association with phenotypic frailty and deficit accumulation in patients with solid tumors across all age groups.

## 2. Methods

A systematic review was conducted using Preferred Reporting Items for Systematic Reviews and Meta-Analysis (PRISMA) guidelines (21).

### 2.1. Eligibility criteria

Inclusion criteria were: (a) published in the English language, (b) molecular measures that correlate with the aging process (hallmarks of aging) (11): telomere attrition, epigenetic alteration, loss of proteostasis, deregulated nutrient sensing, mitochondrial dysfunction, senescence, and inflammation, (c) evaluated phenotypic frailty or deficit accumulation, and (d) measured an association between the biomarker and phenotypic frailty/deficit accumulation. Studies with non-solid tumors and non-human subjects were excluded.

### 2.2. Literature search strategy

A medical librarian (D.C.) conducted electronic database searches of PubMed, Web of Science, and Embase databases of publications from the date of inception to December 08, 2021. Frailty was operationalized as both the phenotypic frailty (7) and deficit accumulation frailty (8) consistent with prior reviews on frailty and biomarkers (15, 16). The search terms included: solid tumors (brain, breast, colon, lung, pancreatic, prostate, and ovarian), biomarkers (cytokines, extracellular vesicles, microRNA, mitochondrial DNA, telomere length, cell senescence markers, inflammageing, epigenetic alterations, mitochondrial dysfunction, and stem cell exhaustion), and outcomes (accelerated aging, frailty, functional decline, and deficit accumulation). The complete search strategy with MeSH terms and Boolean operators for each database is detailed in Supplementary Table S1. References from retrieved reviews and Google Scholar were scanned for additional studies using key search terms.

### 2.3. Data collection

Two reviewers (D.S. and B.P.S.) independently screened titles and abstracts and subsequently full-text articles for study eligibility using the covidence systematic review software (Veritas Health Innovation, Melbourne, Australia). Any incongruencies were resolved upon discussion and consultation with the third researcher (T.S.A.). Two reviewers (D.S. and B.P.S.) completed data abstraction and D.S. reviewed all the final abstracted information. To preserve integrity of the data, the authors kept written communication records of decisions on incongruencies related to data abstraction. Data were extracted using a standardized form for key variables (sample, tumor type, stages, time points, study design, frailty instruments, molecules measured, statistical methods, and key findings).

### 2.4. Risk of bias assessment

Risk of bias assessment was conducted using the National Heart, Lung, and Blood Institute quality assessment tool for observational cohort and cross-sectional studies and a tool for case–control studies (22). The tools consist of 12–14 methodological quality items rated as “yes,” “no,” or “other (cannot determine, not reported, not applicable)” (Supplementary Table S2). The questions evaluate the internal validity of each study, considering the potential risk of biases such as information bias, measurement bias, or outcome bias. The greater the bias (higher number of items rated as “no”), the lower the assigned rating. Reviewers (D.S. and B.P.S.) conducted independent quality assessments. Incongruencies were discussed with the third reviewer’s input (T.S.A.) and concordance was reached upon discussion. To grade the overall quality of the studies, the percentage of items free of bias (items rated as “yes”) out of all possible items was calculated. Studies were assigned overall quality ratings according to the following categories: poor (<50%), fair (≥50% and ≤70%), and good (>70%).

### 2.5. Data analysis

Descriptive statistics were calculated (such as mean, range, and standard deviation) for variable age using either the reported mean or median. Where available, data on race/ethnicity (white vs. non-white) and sex (male vs. female) was extracted.

## 3. Results of synthesis

### 3.1. Study selection

**Figure 1:**
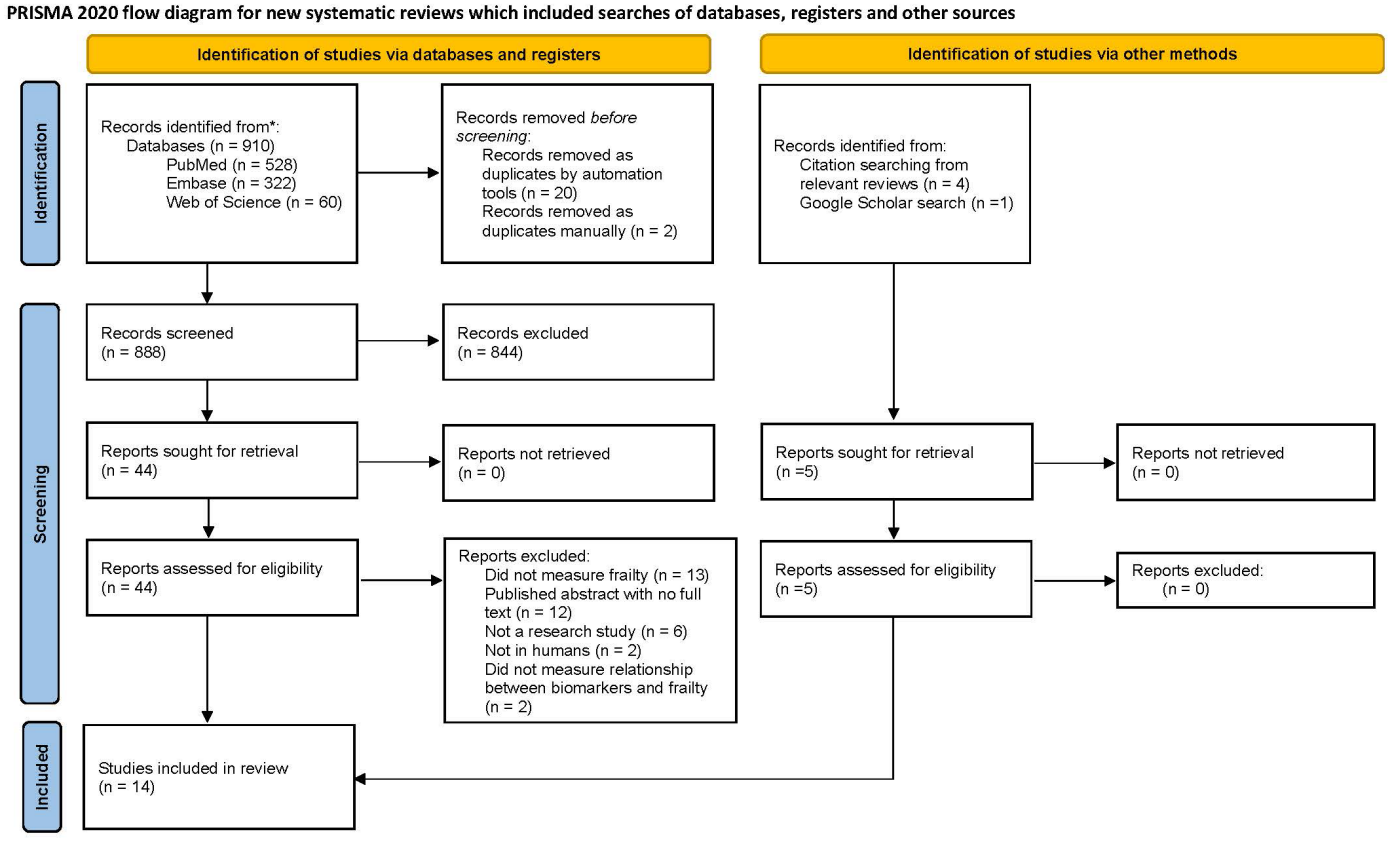
PRISMA flow diagram.

The study selection process is detailed in a PRISMA flow diagram (Figure 1). Briefly, 910 reports were retrieved from the databases. Five additional articles were identified through screening references of relevant reviews and Google Scholar using the search criteria. After removing 19 duplicate reports, search results were uploaded to the covidence software where an additional five reports were identified as duplicates. Two reviewers (D.S. and B.P.S.) independently screened 888 titles and abstracts, of which 844 reports were deemed irrelevant (Supplementary Table S3). Five additional reports were located through Google Scholar and 49 reports were retrieved for full-text review. In total, 14 full-text articles were included in the review. Of the 35 excluded reports, 13 did not measure frailty, 12 were conference abstracts, six were not primary research studies, two were not in human subjects, and two did not measure an association between frailty and biomarkers. Although the study by Falandry and authors (23) did not explicitly use the term “frailty,” the study met the inclusion criteria for measuring “decline in functional reserve” using the geriatric vulnerability score consistent with deficit accumulation definition.

### 3.2. Study and participant characteristics

Characteristics of the included studies are presented in Table 1. All 14 studies were observational study designs. Seven studies were longitudinal cohort studies (23, 26–31), six were cross-sectional (32–37), and one study was a case-control design (38). Across the 14 studies, a total of 2,178 participants were included, with the sample size of each study ranging from 20 to 581. The mean age across all studies was 72 years (standard deviation = 7, range: 53–80 years). Thirteen studies reported information on sex and the distribution was 63% female and 37% male. Four studies reported information on race/ethnicity (28, 29, 37, 38), of which 82% of participants were white and 18% non-white.

**Table 1:** Study and participants characteristics.

Author Year Journal Country	Sample	Tumor Type Stages Time Points	Study design	Frailty instruments	Molecules measured	Statistical methods	Key findings^a^^,^^b^
Brouwers 2015 Aging Belgium	162 participants (old group) **Age**: (median 76) **Sex**: Not reported **Race/ethnicity**: Not reported *Note*: only old group had frailty assessment and is included in this review	**Breast Cancer** **Stages**: Grade I-III Unknown **Time Points**: Pre-treatment	**Cross-sectional**	Balducci score Leuven Oncogeriatric Frailty Score (LOFS) **Balducci Frail criteria**: presence of any of the below criteria (24): ≥85 years ≥1 ADl dependence ≥1 Comorbidity ≥1 Geriatric syndrome **Components of LOFS**: ADL Comorbidities iADL Mental state Nutritional scale	IGF-1 IL-6 MCP-1 RANTES Telomere length	Kruskal-Wallis test (Balducci score) Spearman correlation (LOFS)	**Balducci score**: IL-6 was higher in pre-frail and frail groups **LOFS**: IL-6 also correlated with worse LOFS. **Limitations**: Did not report power analysis Frailty and biomarker measurements are limited to old group alone Did not report post-hoc or multivariate analyses
Buigues 2020 Cancers Spain	39 participants 31% Frail 65% Pre-Frail 17% Robust *Note*: at follow up, 59% had worsening frailty while 41% improved. **Age**: (mean 71.9, SD 9.8) **Sex**: Male 100% **Race/ethnicity**: Not reported	**Prostate Cancer** **Stages**: all stages **Time Points**: During treatment (≥6 months of ADT) Follow-up (~1 year follow-up)	**Prospective longitudinal**	**Fried Frailty Phenotype** **Components of Assessment**: Fatigue Physical activity Walking speed Weakness Weight loss	Basophils CRP Eosinophils Fibrinogen IL-1β IL-6 IL-8 Lymphocytes Monocytes Neutrophils TNF-α	Kruskal–Wallis test followed by multinomial logistic regression controlling for age, gleason score, presence of metastatic disease, prostatectomy, and comorbidity index.	**≥6 months on ADT**^a^ Higher IL-6, IL-8, and lymphocyte count associated with frailty **Follow up**^a^ IL-6 associated with frailty **Progression**^a^ Higher baseline IL-6 and lower lymphocytes associated with frailty progression. **Limitations**: Did not report power analysis Small sample
Bylow 2011 Urology United States	134 participants 63 ADT group 71 Control (non-ADT) group **Age**: ADT group (mean 72.1, SD 7.0) Control group (mean 70.5, SD = 6.3) **Sex**: Male 100% **Race/ethnicity**: **ADT group**: African-American 32% White 67% Other 2% **Control group**: African American 45% White 46% Other 4%	**Prostate Cancer** **Stages**: Not reported **Time Points**: During treatment (≥6 months on ADT) *Note*: control group was post-surgery or radiation without ADT	**Case-Control**	**Fried Frailty Phenotype** **Modified Fried Frailty Phenotype** **Components of Assessment – Fried Frailty Phenotype**: Exhaustion Physical activity Walking speed Weakness Weight loss **Modified Fried Frailty** **Phenotype**: Weight loss replaced by obesity	Albumin CRP Hemoglobin HDL Glucose IL-6 LDL Total cholesterol Triglycerides	T-tests and Fisher’s Exact test	Hemoglobin was lower in ADT compared to non-ADT group. *Note*: ADT group had higher percentage of frail participants using modified FFP. **Limitations**: Did not report power analysis Small sample Did not report multivariate analyses for molecular correlates (hemoglobin)
Corona 2014 Journal of Cellular Physiology Italy	89 participants 49 Fit 23 Unfit 17 Frail **Age**: (median 77, range 70–97) **Sex** Female 100% **Race/ethnicity**: Not reported	**Breast Cancer** **Stages**: Mixed **Time Points**: Pre-treatment	**Cross-sectional**	**Comprehensive Geriatric Assessment (CGA)** **Components of Assessment**: No description of components	40 acylcarnitines 45 aminoacids 150 phospholipids	ANOVA post residual model adjusting for age.	**Unfit &Frail (compared to Fit**)^a^: greater age-adjusted 3-methyl-hystidine **Unfit & Frail (compared to Fit)**^a^: depletion of several age-adjusted sphingolipids and glycerol-phospholipids (SM (OH) C16:1, SM (OH) C24:1, PC aa C32:3, PC aa C34:4, PC aa C36:3, PC aa C36:4, PC aa C38:5, PC ae C32:2, PC ae C34:0, PC ae C34:1, PC ae C34:2, PC ae C34:3, PC ae C36:2, PC ae C36:3, PC ae C36:4, PC ae C36:5, PC ae C38:4, PC ae C38:5, PC ae C42:2, lysoPC a C18:1, lysoPC a C20:4). **Limitations**: Did not report power analysis Small sample
Dalmasso 2018 BioMed Central (BMC) Cancer Belgium	89 participants 46 Chemotherapy (chemo) group 43 Non-chemo group **Age** retrieved from (25): Chemo group (*n* = 57, median = 73.5, range = 70–80) (*n* = 52, median 75, range = 70–90) **Sex**: Female 100% **Race/ethnicity**: Not reported *Note*: Demographic and clinical data was reported on full sample (25)	**Breast Cancer** **Stages**: Locally-advanced Non-metastatic **Time Points**: Inclusion/Pre-treatment (post-surgery and pre-chemo for chemo group) 3 months after inclusion or the day of last chemo for chemo group 1 year after inclusion	**Prospective longitudinal**	Balducci score* **LOFS** Flemish Triage Risk Screening Tool (fTRST)* G8* **Components of LOFS**: ADL Comorbidity Index iADL Mental state Nutritional state *Note*: Balducci, fTRST and G8 components not described.	miR-34a miR-320b miR-378a miR-20a miR-30b miR-106b miR-191 miR-301a miR-374a *Note*: authors also measured telomere length, IL-6, IL-10, TNF-α, RANTES, MCP-1, IGF-1, but did not correlate to frailty.	Spearman correlation followed by multivariable model	Associations with frailty reported at inclusion not separated by groups: **LOFS**^a^: Higher LOFS associated with higher miR374a and lower miR-320b levels **fTRST**^a^: miR-301a negatively correlated with higher frailty **G8**^a^: Lower miR-106b, miR-191, miR320b and higher miR374a served as predictors for total G8 *Note*: No correlations with Balducci score^a^ **Limitations**: Did not report power analysis Small sample
Falandry 2015 Aging France	109 participants **Age**: (median 78, range 70–93) **Sex**: Female 100% **Race/ethnicity**: Not reported	**Ovarian Cancer** **Stages**: FIGO Stage III-IV **Time Points**: Pre-treatment	**Prospective longitudinal**	**Geriatric Vulnerability Score** **Components of Assessment**: ADL iADL HADS Hypoalbumenia Lymphopenia	Telomere length (TL)	Linear regression	GVS ≥3^**a**^ associated with shorter TL group cross-sectionally **Limitations**: Did not report power analysis for effect of TL on GVS
Gilmore 2020 Journal of Geriatric Oncology United States	286 participants 144 Cancer group 142 Non-cancer group **Age**: Cancer group (mean = 60, range 50–76) Non-cancer group (mean 59, range 50–81) **Sex**: Female 100% **Race/ethnicity**: **Cancer group**: 90% White 10% Non-White **Non-cancer group**: 96% White 4% Non-White	**Breast Cancer** **Stages**: I-IIIC Unknown **Time Points**: Pre-treatment (within 7 days prior to chemotherapy) Post-treatment (4 weeks after chemotherapy completion)	**Prospective longitudinal**	**Modified Fried Frailty Phenotype** **Components of Assessment**: Exhaustion Physical activity Walking speed Weakness	IL-6 sTNFRI sTNFRII	Linear regression controlling for pre-chemotherapy frailty scores	**Cancer group**^a^: Greater levels of pre-chemo IL-6, sTNFRI and sTNFRII associated with worse post-chemo frailty in cancer groups *Note*: No associations were found in non-cancer group **Limitations**: Did not report power analysis Cytokines levels are dichotomized due to skewed pre-treatment cytokine distributions
Gilmore 2021 Breast Cancer Research United States	581 Pre-chemotherapy 547 post-chemotherapy 506 six months post-chemotherapy **Age**: (baseline mean 53.4, range 22–81) **Sex**: Female 100% **Race/ethnicity**: White 89% Non-White 11%	**Breast Cancer** Stages: I-IIIC **Time Points**: Pre-treatment (within 7 days) Post-treatment (within 4 weeks after) Post-treatment (6 months after)	**Retrospective longitudinal**	**Modified Fried Frailty Phenotype** **Components of Assessment**: Exhaustion Physical activity Walking speed Weakness	Albumin Hemoglobin Hematocrit Lymphocytes LMR Monocytes Neutrophils NLR Platelets Total WBC	Linear regression analyses controlling for baseline frailty, age, race, marital status, and education, and number of days between blood draw and start or last day of chemo	**Pre-chemo**^a^: Total WBC, neutrophils, NLR associated with pre-chemo frailty **Post-chemo**^a^: Increase from pre-to-post chemo levels of total WBC, neutrophils, and NLR associated with post-chemo (4 weeks after treatment) frailty and in participants who received growth factors with chemo. *Note*: no significant associations from pre-chemo to 6 months post-chemo. **Limitations**: Did not report power analysis
Harneshaug 2019 Journal of Geriatric Oncology Norway	255 participants 127 Frail 128 Non-frail **Age**: (mean = 76.7) Frail group (mean = 77.4) Non-frail (mean 75.5) **Sex**: Female 44% Male 56% **Race/ethnicity**: Not reported	**Mixed Sample**: Breast Prostate Other GI Lung Colorectal Other **Stages**: Localized Locally-advanced Metastatic **Time Points**: Pre-treatment	**Prospective longitudinal**	**Modified GA domains for Balducci’s criteria** **Components of Assessment**: ADL Comorbidity Cognitive function Depressive symptoms Falls Nutritional status Physical function Polypharmacy	GPS (ratio of *Albumin* and *CRP*) IL-6 TNF-α	Logistic regression controlling for tumor type, stage of disease, BMI, use of anti-inflammatory meds.	**GPS 2**^a^ significantly associated with frailty **Limitations**: Heterogenous sample and treatment modalities Higher detection level on ELISAs (higher ULD) Did not report power analysis
Hatse 2014 Public Library of Science (PLOS) One Belgium	*20 Validation Cohort* 10 Older Fit 10 Older Frail *Note*: only validation cohort of older adults received frailty assessment and is included in this review. **Age**: Older fit (mean 78, range 71–83) Older non-fit (mean 78, range 73–91) **Sex**: Female 100% **Race/ethnicity**: Not reported	Breast Cancer **Stages**: I-III **Time Points**: Pre-treatment	**Cross-sectional**	**Balducci** **LOFS** **Balducci**: presence of any of the below criteria (24): ≥85 years ≥1 ADl dependence ≥1 Comorbidity ≥1 geriatric syndrome **Components of LOFS**: ADL iADL Comorbidities Mental state Nutritional scale	miR-320b miR-301a miR-210 miR-21 miR-376a miR-378 miR-374a miR-423-5p miR-20a-3p let-7d miR-191 miR-200c miR-30b-5p miR-140-5p miR-106b	Two group tests with Dunn-Bonferroni correction	No differences between fit and frail groups (Balducci and LOFS) **Limitations**: Did not report power analysis Small sample size Did not report multivariate analyses
Lealdini 2015 Journal of Geriatric Oncology Brazil	52 participants **Age**: (median 72.5, range 65–97) **Sex**: 56% Male 44% Female **Race/ethnicity**: Not reported	**Mixed Sample**: Breast Prostate Stomach Colorectal Head and Neck Lung Endometrial **Stages**: Localized Metastasized **Time Points**: Pre-treatment	**Cross-sectional**	**Edmonton Frailty Scale (EFS)** **Components of Assessment**: ADL Cognition Depression/mood General health status Incontinence Nutrition Physical function Polypharmacy Social support	mGPS, (ratio of *Albumin* and *CRP*)	ANOVA with Bonferroni test or Student T test followed by logistic regression to establish relative risk.	**mGPS 0:** Patients with lower mGPS (0) had lower scores on EFS compared to the mGPS of 2 **mGPS 2**^a^: Patient with mGPS of 2 were 7.5 more likely to have severe frailty **Limitations**: Did not report power analysis Small sample size Did not report multivariate analyses
Navarro-Martinez, 2019 In Urologic Oncology: Seminars and Original Investigations Spain	92 participants 46 Cancer group 46 Control group **Age** (cancer group): (mean 72.2, SD = 9.4) **Sex** (cancer group): Male 100% **Race/ethnicity**: Not reported	**Prostate Cancer** **Stages**: I-IV **Time Points**: During treatment (ADT)	**Cross-sectional**	Fried Frailty Phenotype **Components of Assessment**: Exhaustion Physical activity Walking speed Weakness Weight loss	CRP Creatinine Erythrocytes Fibrinogen Glomerular filtration rate Glucose Hemoglobin IL-1β IL-6 IL-8 Leukocytes Lymphocytes Platelets TNF-α	ANOVA or Kruskal Wallis with posthoc Tukey test for CBC values ANOVA or Kruskal Wallis followed by logistic regression for cytokines	**Cancer group**^a^: higher log IL-6 and fibrinogen were associated with higher odds ratio of being frail **Control group**: significant difference in IL-6, IL-8, CRP with frailty syndrome (Kruskal Wallis). **Limitations**: Demographic data not reported for the control group Did not report post-hoc or multivariate analyses for the control group Did not report power analysis Small sample
Nishijima 2017 Aging United States	133 participants **Age**: (median 74, range 65–92) **Sex**: Female 80% Male 20% **Race/ethnicity**: White 88% Non-White 12%	**Mixed Sample**: Breast Genitourinary Gastrointestinal Lung Other **Stages**: I–IV **Time Points**: Pre-treatment	**Cross-sectional**	**Carolina Frailty Index (CFI)** **Components of Assessment**: iADL Cognitive Function Comorbidities Hearing Falls Medications Mental health Mobility Nutritional status Physical function Social activity Vision	Lymphocytes LMR Neutrophils NLR Monocytes Platelets PLR Total WBC	Spearman correlation test followed by multivariable linear and logistic regression controlling for age, sex, race, education, marital status, cancer type, cancer stage	NLR positively correlated with CFI **Pre-frail ***vs*** frail**^a^: Patients with NLR at top teritles had higher odds of being pre-frail and frail. **Limitations**: Did not report power analysis
Ronning 2010 Age and Aging Norway	137 participants **Age**: (median 80 range 70–94) **Sex**: Female 55% Male 45% **Race/ethnicity**: Not reported	**Colorectal Cancer** **Stages**: Localized Regional lymph Node metastases Distant metastasis Not determined **Time Points**: Pre-treatment	**Prospective longitudinal**	**Fried Frailty phenotype** **CGA frailty categories** **Components of** **Fried frailty phenotype**: Exhaustion Walking speed Weakness Weight loss Low physical activity **Components of CGA frailty**: ADL Comorbidities Cognitive function Depression Functional Dependence Nutritional Status Polypharmacy	CRP IL-6 TNF-a D-dimer	Kruskal-Wallis followed by Mann–Whitney U test with Bonferroni correction	**FFP results**^b^: CRP and IL-6 were higher in frail versus pre-frail groups for both frailty phenotypes TNF-α levels were also significantly higher in pre-frail versus robust group **CGA results**^**b**^: CRP and IL-6 were higher in intermediate versus fit and frail versus intermediate groups TNF-α was significantly higher in frail than intermediate group **Limitations**: Did not report power analysis Did not report multivariate analyses for frailty outcomes Small sample

ADL, activities of daily living, ADT, androgen deprivation therapy, iADL, instrumental ADL; α, alpha; CFI, Carolina frailty index; IL, interleukin; IGF, insulin-like growth factor 1; MCP-1, monocyte chemoattractant protein-1; RANTES, regulated upon activation, normal T cell expressed and secreted; fTRST, Flemish triage risk screening tool; HADS, Hospital anxiety depression scale; EFS, edmonton frailty scale; CGA, comprehensive geriatric assessment; GA, geriatric assessment; TNF-α, tumor necrosis factor-α; SD, standard deviation; TST, time since treatment; CRP, C-reactive protein; HDL, high density lipoprotein; LDL, low density lipoprotein; LOF, Leuven Oncogeriatric Frailty Score; miRNA, micro RNA; GPS, Glasgow prognostic score; mGPS, modified GPS; sTNFR I, soluble TNF receptor I; LMR, lymphocyte to monocyte ratio; NLR, neutrophil to lymphocyte ratio; PLR, platelet to lymphocyte ratio; WBC, white blood cells; ECOG-PS, Eastern cooperative oncology group performance scale.

a Denotes findings that remained significant after inclusion in multivariate analyses.

b Denotes findings that remained significant after post hoc analyses.

The most commonly studied solid tumor was breast (*n* = 6, 43%) (27–29, 32–34), followed by prostate tumor (*n* = 3, 21%) (26, 36, 38) (Table 1). Stages of cancer varied greatly ranging from stage I to IV (or localized to metastasized) and most studies were initiated at pre-treatment (i.e., at the diagnosis, pre-inclusion, prior to surgery or adjuvant treatments) (*n* = 11) (23, 27–35, 37) (Table 1). Among studies which included participants on treatment (*n* = 6, 43%), three studies in prostate cancer (*n* = 3, 21%) had patients receiving ADT and three studies with participants with breast cancer had patients on adjuvant or neoadjuvant chemotherapy and/or endocrine treatment.

**Figure 2:**
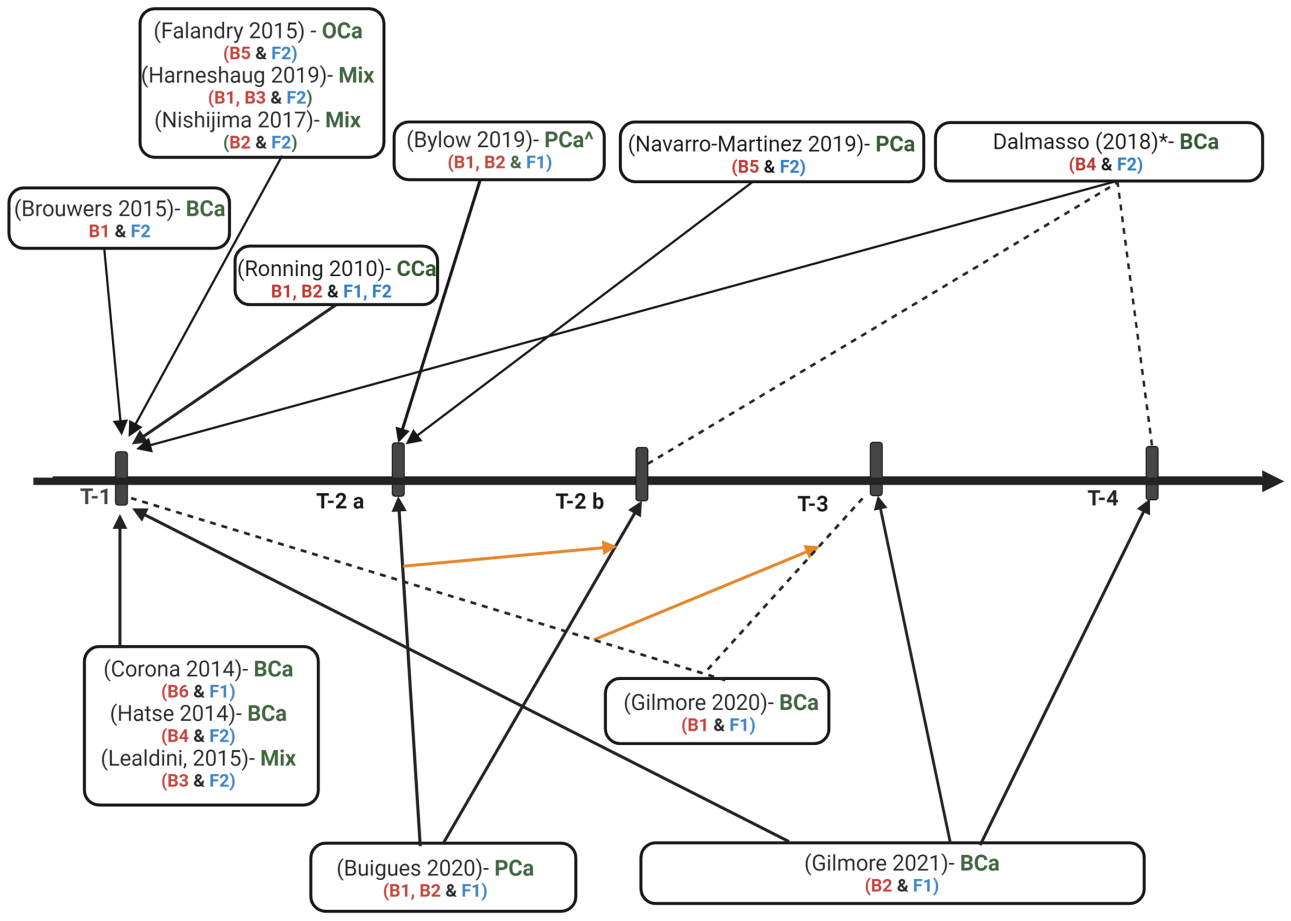
Time-points for frailty and biomarker assessments. T-1 = pre-treatment (pre-surgery or post-surgery but pre-adjunctive therapy), T-2 a = during treatment (T-2 b = follow up since treatment beginning but not post-treatment), T-3 = 4 weeks post-treatment, T-4 = 6 months or greater post-treatment, BCa = breast cancer, PCa = prostate cancer, mix = mix solid tumors, Oca = ovarian cancer, CCa = colorectal cancer, B1 = cytokines, cytokine receptors, and acute phase proteins, B2 = molecules from complete blood count, lipid panel, or chemistry panel, B3 = glasgow prognostic score (GPS), B4 = micro RNAs, B5 = telomere length, B6 = metabolomics, F1 = physical frailty phenotype measured by fried frailty phenotype tool, F2 = deficit accumulation or geriatric vulnerability based frailty measured by geriatric assessment (GA) or GA domains (Balducci score, Leuven Oncogeriatric Frailty Score, Comprehensive Geriatric Assessment, Flemish Triage Risk Screening Tool, geriatric vulnerability score, Edmonton Frailty Scale, and Carolina Frailty Index). * = timeline is the same for cancer group with chemotherapy and without. ^control group with history of PCa, post-surgery or radiation therapy. Biomarker levels, frailty scores, and the association was measured between the two, pre-treatment biomarkers were associated with post-treatment frailty scores, biomarker levels and frailty scores were measured but did not evaluate the association between the two.

Evaluation of the association between biomarker levels and frailty occurred cross-sectionally in 11 studies (Figure 2). Two studies reported an evaluation of the association between the biomarker and frailty at multiple time points (26, 29). Buigues and authors (26) evaluated the association during treatment (six months or greater on treatment) and at one-year follow-up, notably, authors do not indicate one-year follow-up as post-treatment. Another study (29) evaluated the association of pre-treatment cell counts with pre-treatment frailty scores and an increase in cell counts from pre-treatment to four weeks or six months post-treatment with post-treatment frailty scores. Gilmore and authors (28) evaluated pre-treatment levels of cytokines as predictors of post-treatment frailty, but not at each time point.

### 3.3. Assessments of phenotypic frailty/deficit accumulation

Frailty measurements varied greatly across the 14 studies. Fried frailty phenotype (FFP) was the most common instrument used (*n* = 6). The instrument’s prespecified criteria were applied across four studies (26, 31, 36, 38), where “frail” was defined as the presence of three or more components, “pre-frail” with one to two components, and “robust” with zero components (7). However, three studies used a modified version of the FFP, where two reports did not include unintended weight loss (28, 29) and one study replaced unintended weight loss with obesity (38).

Eight studies measured frailty as a deficit accumulation index or geriatric vulnerability scores using the Leuven Oncogeriatric Frailty Score (27, 32, 34), Balducci criteria (27, 30, 32, 34), Flemish Triage Risk Screening Tool (fTRST) (27), G8 (27), geriatric assessment domains (30, 31), the geriatric vulnerability score (23), the Edmonton frailty scale (35), and the Carolina frailty index (37). One study (23) used geriatric assessment domains that also included hypoalbuminemia and lymphopenia as two additional vulnerabilities calculated into the total geriatric vulnerability score. Two studies did not report which domains were assessed in comprehensive geriatric assessment (CGA) (33), Flemish Triage Risk Screening Tool, Balducci, or the G8 (27).

Four studies used multiple deficit accumulation frailty tools (Table 1). Two reports used the Balducci frailty category together with Leuven Oncogeriatric Frailty Score (32, 34); whereas, one study added the Flemish Triage Risk Screening Tool and G8, in addition to Balducci and Leuven Oncogeriatric Frailty Score (27). Although the frailty scores differed based on the instrument applied to either continuous scoring and/or frailty group categories, the authors reported frailty scores and their association with biomarkers across all the tools used (27, 31, 32, 34).

### 3.4. Blood-based biomarkers

**Figure 3:**
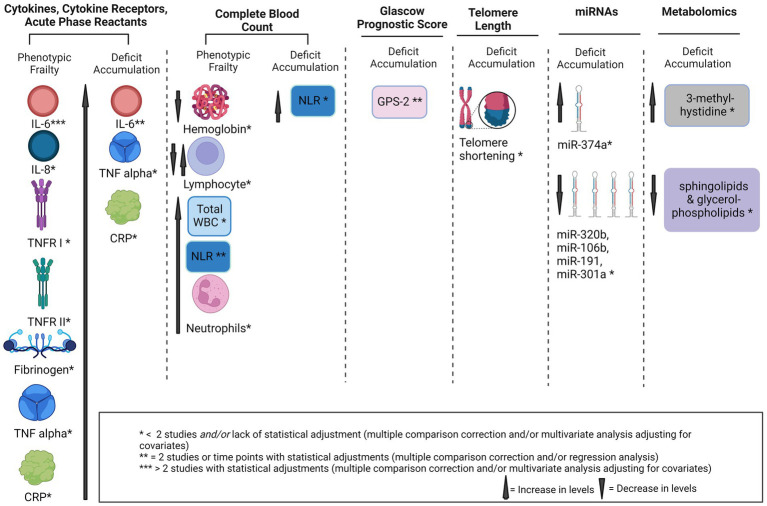
Potential biomarkers of frailty in solid tumors. IL, interleukin, GA, geriatric assessment, TNF-α, tumor necrosis factor-α, CRP, C-reactive protein, miRNA, micro RNA, GPS, glasgow prognostic score, sTNFR I, soluble TNF receptor I, NLR, neutrophil to lymphocyte ratio, WBC, white blood cells.

Peripherally circulating blood-based markers were measured across all 14 studies to evaluate their association with frailty. Only one report found no significant association with frailty in any of the markers measured (34). The statistically significant findings (*p* values < 0.05) identified in this review are presented below and categorized into six categories: cytokines/cytokine receptors and acute phase reactants; complete blood count; Glasgow Prognostic Score; microRNAs; telomere length; and metabolomics (Figure 3).

### 3.5. Cytokines, cytokine receptors, and acute phase reactants

Cytokines, cytokine receptors, and acute phase reactants associated with frailty included: interleukin (IL)-6, IL-8, tumor necrosis factor (TNF)-α, soluble TNF receptor I (TNFR I), soluble TNFR II, C-reactive protein (CRP), and fibrinogen (Figure 3). Results are separated by frailty construct and time points for frailty measurements.

#### 3.5.1. Phenotypic frailty: pre-treatment

Pre-treatment levels of IL-6, TNF-α, and CRP were significantly associated with increased phenotypic frailty in colorectal tumors (31). Of note, while Gilmore and authors (28) measured pre-treatment levels of IL-6, the associations were tested with post-treatment frailty scores, therefore, the results are described below.

#### 3.5.2. Phenotypic frailty: during treatment

Higher levels of IL-6 were associated with higher phenotypic frailty in prostate cancer during androgen deprivation treatment (ADT) (26, 36) and at one-year follow-up (26). However, IL-6 and CRP were not associated with higher phenotypic frailty on six months of ADT in another prostate cancer cohort (38). While Buigues and authors (26) found that higher levels of IL-8 were associated with frailty at inclusion (six months or greater on ADT), IL-8 was no longer associated with frailty at one-year follow-up from inclusion. Similarly, another study (36) reported null findings during treatment, whose cohort had an average of 106 months from diagnosis. The two cohorts reported mixed findings on the association of fibrinogen with phenotypic frailty, where Navarro-Martinez and authors (36) found that higher levels of fibrinogen were associated with frailty, but Buigues and authors (26) reported null findings. Findings were also null for CRP, IL-1β, and TNF-α in these two prostate cancer cohorts (26, 36).

Of note, while Navarro-Martinez and authors (36) also included a non-cancer control group, the adjusted results with posthoc analysis were reported for the ADT group but not the control group, making their comparison challenging. Unadjusted higher levels of CRP, IL-6, and IL-8 were associated with greater frailty in the non-cancer control group.

#### 3.5.3. Phenotypic frailty: post-treatment

Pre-treatment levels of IL-6, soluble TNFR I and II were significantly associated with four weeks post-treatment phenotypic frailty in the breast cancer group. Notably, no associations were found with any of the biomarkers in the age-matched non-cancer group (28).

#### 3.5.4. Deficit accumulation frailty: pre-treatment

In pre-treatment studies, IL-6 was significantly associated with increased deficit accumulation frailty (Balducci and Leuven Oncogeriatric Frailty Score) in breast cancer (32). In patients with colorectal cancer (31), authors reported increasing trends of IL-6 and CRP across stratified levels of deficit accumulation frailty (geriatric assessment domains) ranging from fit to frail. Authors also found higher levels of TNF-α in frail versus intermediate groups (31). However, no association between IL-6 or TNF-α and greater frailty (Balducci criteria) was found in mixed solid tumors (30).

### 3.6. Complete blood count

Five studies investigated the association between markers of complete blood count and frailty (26, 29, 36–38).

#### 3.6.1. Phenotypic frailty: pre-treatment

At pre-treatment, greater total white blood cell (WBC) count, neutrophils, and neutrophil-lymphocyte ratio (NLR) were associated with phenotypic frailty in patients with breast cancer (29). However, hemoglobin was not associated with frailty in the same cohort (29).

#### 3.6.2. Phenotypic frailty: during treatment

During ADT, Buigues and authors (26) found that a higher lymphocyte count was associated with significant odds of being frail in patients six months or greater on ADT. In contrast, a lower lymphocyte count was associated with frailty progression at a one-year follow-up (26). In another prostate cancer cohort, lower hemoglobin was found in the ADT group compared to the non-ADT control group (38). The authors did not find a significant association with other cell markers. Similarly, total WBC, leukocyte counts, or hemoglobin did not predict frailty states in another study (36).

#### 3.6.3. Phenotypic frailty: post-treatment

Pre-to post-treatment increases in WBC, neutrophils, and NLR predicted greater four-week post-treatment frailty in breast cancer, however, none of these markers were significant predictors of six months post-treatment frailty (29). Null findings were reported for hemoglobin or other cell markers in breast cancer post-treatment (29).

#### 3.6.4. Deficit accumulation frailty: pre-treatment

One study was found to evaluate complete blood count with deficit accumulation frailty at pre-treatment in mixed tumor types. Authors (37) found that greater NLR was associated with frailty (Carolina Frailty Index), however, they found null findings in total WBC or other cell counts.

### 3.7. Glasgow prognostic score

#### 3.7.1. Deficit accumulation frailty: pre-treatment

Glasgow Prognostic Score (GPS), the ratio between CRP and albumin, was tested as a biomarker of frailty in two studies (30, 35). Both studies included patients with mixed solid tumors in the pre-treatment phase (30, 35) and found GPS 2 (elevated CRP and hypoalbuminemia) to be significantly associated with deficit accumulation frailty (Balducci criteria and Edmonton Frailty Scale).

### 3.8. MicroRNAs

#### 3.8.1. Deficit accumulation frailty: pre-treatment

Two studies evaluated microRNAs (miRNAs) as biomarkers of deficit accumulation frailty (Balducci, Leuven Oncogeriatric Frailty Score, Flemish Triage Risk Screening Tool, G8) in patients with breast cancer (27, 34) at pre-treatment. Dalmasso and authors (27) found that higher miR374a and lower miR-320b levels were associated with lower frailty using the Leuven Oncogeriatric Frailty Score and levels of miR-301a negatively correlated with frailty using Flemish Triage Risk Screening Tool scores. In addition, lower miR-106b, miR-191, miR-320b, and higher miR-374a emerged as independent predictors of deficit accumulation frailty using G8 (27). In comparison, Hatse and authors (34) reported null findings for 15 evaluated miRNAs and deficit accumulation frailty (Balducci, Leuven Oncogeriatric Frailty Scores).

### 3.9. Telomere length

#### 3.9.1. Deficit accumulation frailty: pre-treatment

Two studies evaluated the relationship between telomere length and deficit accumulation frailty (Balducci, Leuven Oncogeriatric Frailty Score, geriatric vulnerability score) at pre-treatment (23, 32). In patients with ovarian cancer, shorter telomere length was associated with a geriatric vulnerability score ≥3 (23). However, findings were null in patients with breast cancer (32).

### 3.10. Metabolomics

#### 3.10.1. Deficit accumulation frailty: pre-treatment

The search yielded only one study that evaluated a metabolomic profile of different amino acids, acylcarnitines, and phospholipids as biomarkers of deficit accumulation frailty (comprehensive geriatric assessment) in patients with breast cancer (33). The authors found greater age-adjusted ß3-methyl-hystidine levels in unfit and frail groups compared to the fit group. Similarly, they found depletion of several sphingolipids and glycerol-phospholipids in unfit and frail groups compared to fit (Table 1).

### 3.11. Risk of bias and quality assessment

The risk of bias and quality assessment results are presented in Table 2. Interrater reliability for cross-sectional and cohort studies between the two reviewers was 83 and 67% for the case–control study. Six studies were rated as good (23, 26, 27, 29–31), while the remaining eight were rated as fair. Several areas of potential bias in this body of literature were identified: participant sampling procedures, power analyses, measurement biases, instrumentation, and statistical methods. Most of the cohort studies (12/13) reported selecting participants during the same period and applying inclusion criteria uniformly (23, 26–35, 37). One study selected prostate cancer group undergoing ADT and the control group from nursing home facilities, thus the two groups differed in diagnosis, active treatment, clinical setting and therefore were rated as dissimilar or “no” for the criterion on sampling methodology (question 4) (36).

**Table 2:** Quality assessment.

Author and year	Q1	Q2	Q3	Q4	Q5	Q6	Q7	Q8	Q9	Q10	Q11	Q12	Q13	Q14	# of items free of bias	% of items free of bias	Qualitative rating
**Observational cohort** ^a^																	
Buigues et al. 2020	Yes	Yes	Yes	Yes	No	Yes	Yes	Yes	Yes	Yes	Yes	CD	Yes	Yes	12	86	Good
Dalmasso et al. 2018	Yes	Yes	Yes	Yes	No	Yes	Yes	Yes	Yes	Yes	No	CD	Yes	Yes	11	79	Good
Falandry et al. 2015	Yes	Yes	Yes	Yes	No	Yes	Yes	Yes	Yes	No	Yes	CD	Yes	Yes	11	79	Good
Gilmore et al. 2020	Yes	Yes	NR	Yes	No	Yes	Yes	Yes	Yes	No	No	CD	Yes	Yes	9	64	Fair
Gilmore et al. 2021	Yes	Yes	NR	Yes	No	Yes	Yes	Yes	Yes	Yes	No	CD	Yes	Yes	10	71	Good
Harneshaug et al. 2019	Yes	Yes	Yes	Yes	No	Yes	Yes	Yes	Yes	No	No	CD	Yes	Yes	10	71	Good
Ronning et al. 2010	Yes	Yes	Yes	Yes	No	Yes	Yes	Yes	Yes	No	Yes	CD	Yes	No	10	71	Good
**Cross-sectional** ^a^																	
Brouwers et al. 2015	Yes	Yes	NR	Yes	No	No*	No*	Yes	Yes	No*	No	CD	No*	No	5	50	Fair
Corona et al. 2014	Yes	Yes	Yes	Yes	No	No*	No*	Yes	Yes	No*	No	CD	No*	No	6	60	Fair
Hatse et al. 2014	Yes	Yes	Yes	Yes	No	No*	No*	Yes	Yes	No*	No	CD	No*	No	6	60	Fair
Lealdini et al. 2015	Yes	Yes	NR	Yes	No	No*	No*	Yes	Yes	No*	Yes	CD	No*	No	6	60	Fair
Navarro-Martinez et al. 2019	Yes	Yes	NR	No	No	No*	No*	Yes	Yes	No*	Yes	CD	No*	CD	5	50	Fair
Nishijima et al. 2017	Yes	Yes	NR	Yes	No	No*	No*	Yes	Yes	No*	Yes	CD	No*	Yes	7	70	Fair
**Case–control** ^b^																	
	**Q1**	**Q2**	**Q3**	**Q4**	**Q5**	**Q6**	**Q7**	**Q8**	**Q9**	**Q10**	**Q11**	**Q12**	**# of items free of bias**	**% of items free of bias**	**Qualitative rating**		
Bylow et al. 2011	Yes	Yes	No	Yes	Yes	Yes	CD	CD	Yes	Yes	CD	No	7	58	Fair		

*Questions that were not applicable to cross-sectional design studies were not counted toward overall score.

a Cohort and cross-sectional studies were evaluated using NIH quality assessment tool for observational cohort and cross-sectional.

b Case-control study was evaluated using the quality assessment tool for case-control studies.

c Cancer group had multivariate analyses but not the control group.

None of the included studies reported sample size justification through power analysis. Among the observational longitudinal cohorts, four studies (23, 28, 30, 31) measured biomarkers at only one-time points, while reporting longitudinal outcomes such as survival. Thus, these four studies received a “no” rating for the repeated exposure measurement criterion. Among the evaluation of outcome (frailty), over half of the reports either did not use previously validated cut-off scores or modified existing tools without prior validation. All studies were either missing blinding procedures or failed to report them, thus potential risk for bias could not be determined. Only seven studies (23, 26–30, 37) controlled for confounders through multivariate analyses. Lack of multivariate analyses may introduce potential confounding bias in overestimation or underestimation of markers’ impact on frailty. In the single case-control study, the investigators did not provide sample size justification or blinding procedures (38). The investigators also did not specify if concurrent controls were used or if 100% of eligible cases were recruited, thus, it was unclear if participants in the control group were recruited at the same time as cases. Measures of association or effect sizes were not reported or partially reported in seven included studies (28, 31–33, 35, 36, 38; Supplementary Table S4).

## 4. Discussion

In our review evaluating biomarkers of frailty in solid tumors, we identified IL-6, NLR, and GPS 2 as potential biomarkers of frailty found across two or more studies. To our knowledge, this is the first systematic review evaluating existing biomarkers of frailty in patients with solid tumors. While the inclusion criteria included all solid tumors, the search yielded findings in breast, prostate, mixed solid tumors, ovarian, and colorectal cancers with no studies identified in brain, pancreatic, lung, or other solid organ cancers. The included studies used two distinct frailty constructs, phenotypic frailty and deficit accumulation, which are described in prior literature (10, 39, 40). These distinct frailty paradigms make synthetization challenging. We found that biomarkers were most frequently evaluated and associated with phenotypic and deficit accumulation frailty at pre-treatment although associations were found across the cancer continuum.

Inflammatory molecules were most frequently measured and significantly associated with phenotypic and deficit accumulation frailty, on par with prior reviews that evaluated biomarkers of frailty primarily in older individuals with mixed diagnoses (15–18). Cytokines, cytokine receptors, and acute phase reactants were among the most commonly measured, perhaps due to their role as modulators of cell-to-cell communication in inflammatory responses and cancer biology (41, 42).

Five studies reported elevated levels of IL-6, a pleiotropic pro-inflammatory cytokine, in patients with higher phenotypic and deficit accumulation frailty across the breast, prostate, and colorectal tumors (26, 28, 31, 32, 36). Elevated levels of IL-6 have been documented in aging, cancer progression, and the development of cancer cachexia (43). Moreover, IL-6 can be elevated in both acute and chronic immune responses by exerting stimulatory effects on T and B cells and producing acute-phase reactants (44). Included studies reported higher levels of IL-6 associated with phenotypic and deficit accumulation frailty evaluated at pre-treatment, during treatment, and four weeks post-treatment. However, two studies reported null findings: six months on ADT with phenotypic frailty (38) and with pre-treatment deficit accumulation frailty (30). Bylow and authors (38) did not find significance when comparing their ADT group (more frail group) to their non-ADT group (less frail group), which suggested that ADT-associated frailty may not be related to circulating increases in IL-6. Harneshaug and authors (30) found a significant association with pre-treatment deficit accumulation frailty, but the findings were null after adjustment for confounders. That coupled with the absence of multivariate analyses in the studies with positive findings (31, 32), suggests elevated IL-6 may be related to the clinical confounders and analytical adjustments are necessary to parse the relationships. IL-6, as a multifaceted cytokine, has been shown to be elevated in chronic inflammatory states such as aging, cancer, obesity (43, 45) and plays a role in underlying pathology of worsening disease states (18). We hypothesize that elevated levels of IL-6 in worsening frailty may be explained by a greater number of inflammation related symptoms and conditions (18).

IL-8, a pro-inflammatory chemokine, was evaluated in two of the studies (26, 36) and found to serve as a correlate of frailty during treatment (six months or greater on ADT), but not at one-year follow-up (26). In contrast, null findings were reported during treatment in another prostate cancer group (36). Although both studies (26, 36) studied IL-8 and phenotypic frailty during ADT, their discrepant findings may be owed to their analytical methods: namely, post-hoc statistical adjustment versus multivariate regression. Additionally, Navarro-Martinez and authors (36) did not report a list of variables included in the multinomial regression which made it difficult to compare to Buigues and authors (26). Thus, although IL-8 has been postulated to rise during ADT (46), the evidence remains inconclusive and is limited by these two studies with varying methods and small sample sizes (26, 36). A possible explanation for the association between IL-8 and frailty could be that frail individuals may be more susceptible to acute inflammatory response during treatment, which may manifest as reduced physical activity and increased frailty symptomology (2).

TNF-α was evaluated in four reports (26, 30, 31, 36) and found to associate with pre-treatment phenotypic and deficit accumulation frailty in colorectal cancer (31). The associations were null in pre-treatment deficit accumulation in mixed tumors (30) or during treatment with phenotypic frailty in prostate tumors (26, 36). The incongruencies for phenotypic frailty may relate to the heterogeneity in tumor types and time from treatment: pre-treatment (31) versus during treatment (26, 36). Findings were also incongruent for pre-treatment deficit accumulation frailty, where one study (31) found higher levels of TNF-α in the frail group, but another (30) had null findings after adjustment for confounding variables in the multivariate analysis. Importantly, the study by Ronning and authors (31) lacked multivariate adjustments altogether. Soluble TNFR I and II, members of the TNF superfamily, were measured only in one study with post-treatment phenotypic frailty, and findings, albeit significant, are exploratory and thus warrant additional corroborations (28). Thus, the relationships between phenotypic and deficit accumulation frailty severity and TNF-α, soluble TNFR I and II remain unclear.

Consistent with a previous meta-analysis of frailty biomarkers in primarily non-cancer diagnoses of older adults (18), CRP and fibrinogen emerged as correlates of phenotypic and deficit accumulation frailty at pre-treatment (31) and with phenotypic frailty during treatment (36). Importantly, CRP was not significant in three studies of patients with prostate tumors on ADT (26, 36, 38), whereas fibrinogen was not significant in one report (26). The finding by Ronning and authors (31) of elevated pre-treatment CRP in frail groups may correlate with tumor-mediated inflammatory response (47). However, further extrapolation would yield ambiguous conclusions, given the cross-sectional time points and lack of pre-treatment levels for comparison across all four reports. Collectively, findings for IL-6, IL-8, TNF-α, CRP, and fibrinogen suggest that higher levels of pro-inflammatory cytokines and acute phase reactants may play a role in frailty states in patients with solid tumors. Increased levels of inflammation markers may be related to cancer and its treatment effects on frail and pre-frail cancer survivors. Additionally, although we did not restrict the age of the participants for the inclusion criteria in this review, the average age across 14 studies was 72 years. Older age has a linear relationship with low grade chronic inflammation and is subsequently associated with increased comorbidity and higher vulnerability to disease, which may, in turn, be manifested as frailty signs/symptoms such as weakness, decreased physical activity, and exhaustion (16, 18).

Perturbations in neutrophils, lymphocytes, total WBC, and NLR may be related to both tumor promoting and immune suppressive roles associated with poor outcomes in solid tumors (48–52). Across the five studies that evaluated markers of complete blood counts, NLR, a quotient of neutrophil and lymphocyte counts, emerged as a significant predictor of pre-treatment and post-treatment phenotypic frailty in breast cancer (29) and pre-treatment deficit accumulation frailty in mixed tumor types (37). High NLR has been shown to associate with greater phenotypic and deficit accumulation frailty in cancer survivors, patients with cardiovascular disease, and community dwelling older adults (15). Notably, the study by Gilmore and authors (29) found associations between increased NLR, total WBC, neutrophils and frailty scores pre-chemotherapy and four weeks post-chemotherapy; however these markers and frailty scores returned to baseline six months post treatment. We hypothesize the observed elevations in NLR, total WBC, neutrophils and their association with increased frailty symptomology may be related to an acute inflammatory response to cancer pathology and treatment effects.

Higher lymphocyte levels were associated with phenotypic frailty during treatment in patients on ADT six months or greater prior to inclusion; however, when evaluating progression to frailty at one year follow-up, lower lymphocyte levels associated with the likelihood of being frail (26). The discrepancy may relate to the frailty scores at inclusion versus one year follow-up, reflecting the long-term effect of ADT on frailty progression and the potential effect on lymphopoiesis (53). Additionally, decreased physical activity (a component of frailty phenotype) was previously reported to be associated with lower lymphocyte counts, whereas increased physical activity was associated with higher lymphocyte counts. Prior scoping review also documented an association between lower lymphocyte counts in the presence of frailty (15). Lymphocyte counts did not associate with phenotypic frailty pre-or post-treatment in the breast (29) or pre-treatment deficit accumulation in mixed solid tumors (37). The discrepant findings across the three studies may be related to heterogeneity in the types of solid tumors and frailty definitions.

Hemoglobin, a marker of anemia, was evaluated in three studies and was found to be associated with phenotypic frailty in patients with prostate tumors six months on ADT (38). However, this association was not corroborated by the other two reports with phenotypic frailty before and during treatment in neither prostate nor breast tumors (29, 36). The association found by Bylow and authors (38) may relate to the inverse relationship between androgen deprivation treatment and hemoglobin levels, where treatment may cause decline in hemoglobin (53). ADT-related lower hemoglobin (i.e., anemia) has been associated with symptoms such as fatigue and decreased activity (53), thus, it is plausible that lower hemoglobin in the study by Bylow and authors (38) may be related to the exhaustion and decreased physical activity symptoms/components of the phenotypic frailty.

GPS, the ratio between CRP and albumin, has been extensively validated as a biomarker of poor prognosis in cancer (54). GPS includes scores of 0, 1, 2, with scores ≥2 signifying both hypoalbuminemia (<35 g/L) and elevated CRP levels (>10 mg/L) (54). While CRP is a pro-inflammatory molecule, hypoalbuminemia reflects poor nutritional status associated with increased mortality in patients with cancer (55). In this review, two reports found GPS 2 to significantly associate with deficit accumulation frailty at pre-treatment with moderate to excellent specificity (30, 35). Previously, GPS 2 was shown to associate with cancer-related cachexia, weight loss, and poor performance status (54, 56); however, the two reports which evaluated frailty with GPS in the present review did not measure weight loss. Additionally, pronounced inflammatory response induces hypoalbuminemia (57), and the aging process, itself has been linked to lower levels of albumin (58). Because the patients included in the aforementioned reports were >70 years of age with mixed solid tumors, stages, and treatments (30, 35), we hypothesize that GPS 2 (i.e., elevated CRP and hypoalbuminemia) may be related to the physiological processes underlying cancer, aging, and geriatric vulnerabilities which comprised the deficit accumulation frailty scores.

Epigenetic alterations are another hallmark of aging (11) and are causally related to miRNA dysregulations in cancer (59). Among the reports included, two studies evaluated aging-related miRNAs as molecular correlates of pre-treatment deficit accumulation frailty. Dalmasso and authors (27) found an association between higher levels of aging-related miR-320b and higher frailty using the Leuven Oncogeriatric Frailty Score (LOFS) but not with the Balducci score. They also report an inverse relationship with G8 scores and miR-106b, miR-191, and miR320b, suggesting lower levels are associated with higher scores. Given the established link between the miRNAs with aging process (11) and their dysregulation in cancer biology (59), we hypothesize the exploratory findings reported by Dalmasso and colleagues (27) may be related to the older age of participants included (median age > 74 years), cancer biology, and amalgamation of geriatric deficits comprising LOFS and G8. In contrast, a report by Hatse and authors (34) did not find these associations in a smaller cohort of older frail (*n* = 10) patients with breast cancer. The validation study by Hatse and authors (34) was used as pilot validation cohort and nonsignificant findings in relation to frailty may relate to the smaller sample size. Additional studies are warranted to further extrapolate relationship between aging miRNAs and phenotypic/deficit accumulation frailty phenotypes.

Telomere length also associates with pre-treatment deficit accumulation frailty. Telomeres are nucleoprotein structures located at the chromosomal ends and telomere length attrition is attributed to telomerase deficiency and lack of DNA repair (11, 60). Telomere dysfunction, linked to cell senescence, apoptosis (11), and tissue inflammation, gives rise to diseases with inflammatory components such as cancer (60). While shorter telomere length was associated with greater pre-treatment deficit accumulation frailty in patients with ovarian cancer (23), findings were null in patients with breast cancer (32). This discrepancy may be due to the varying geriatric domains that comprise the geriatric vulnerability score (23), Baducci, and the Leuven Oncogeriatric Frailty Score (32). Given the previously established bidirectional link between inflammation and telomere attrition (61), it is plausible that the shorter telomere length found in the ovarian cancer cohort (23) relates to inflammation and hypoalbuminemia components of GVS. Conversely, shortened telomere length may also relate to differences in stages of cancer: stages I–III in the breast cancer cohort (32) compared to stages III–IV in the ovarian cancer cohort (23). The evidence presented here does not support the extrapolation of the link between shorter telomere length and frailty state in solid tumors. Additional studies investigating telomere capacity as biomarkers of frailty are needed to compare frail versus non-frail cohorts with similar age, disease, and treatment before this finding can be confirmed.

Only one study incorporated a global approach by using metabolomics to investigate a comprehensive profile of amino acids, acylcarnitines, and phospholipids in association with pre-treatment deficit accumulation frailty (33). Metabolomics is a powerful tool that enables researchers to profile endogenous metabolites and metabolic pathways underlying disease (62, 63). Researchers propose that metabolomics may capture the multifactorial frailty profiles (63). Corona and authors (33) found that age-adjusted 3-methylhistidine (3MHis) was elevated and levels of sphingolipids and glycerophospholipids were decreased in frail patients with breast cancer. Higher 3MHis relates to skeletal muscle loss observed with older age (64) in healthy adults, whereas the dysregulation of sphingolipids and glycerophospholipids relates to the progression of metabolic disease (65). A recent study evaluating the metabolomic profile of frailty phenotype in healthy older adults stratified by gender identified modulators of prefrailty phosphatidylglycerol (26:1) and dimethyloxazale for men and threonine, fructose, mannose, dihydroxyphenyl acetic acid, and 2,4-aminobutyric acid for women (66). While the metabolites in the two studies differed, the metabolomics results suggest perturbations in the metabolites may be associated with frailty, but further validation in each solid tumor type is needed.

Interpreting these results requires caution due to several limitations. First, the studies’ frailty instruments measured different constructs of frailty, including phenotypic versus deficit accumulation frailty. Our findings here highlight variations in the constructs, operationalization, and instruments used to assess frailty, of which some were validated. These issues are echoed by findings from previous reviews (40, 67) and a clinician survey (68) of limited validity across instruments and different operationalizations of the frailty concept. Modification of existing tools and lack of validity and reliability support for novel tools collectively threaten the internal and external validity of findings in this body of literature.

Second, great heterogeneity in analysis was found across studies. While some reports incorporated multiple logistic regression, others used bivariate correlations and tests by three groups (e.g., Kruskal-Wallis) to draw associations between the molecular correlates and frailty scores. We found that several studies did not report multiple comparison corrections and adjustments for significant covariates, which would introduce type II error and the potential for multicollinearity. The variation in statistical approach makes it difficult to synthesize findings across studies.

Third, included studies did not report power analyses, although the majority reported smaller sample sizes. This indicates that the evidence is, at this point, largely exploratory and warrants larger corroborative investigations. Moreover, only half the included studies reported measures of association/effect sizes for statistically significant results, which limits our ability to comment on clinically meaningful effect. Future investigations would benefit from reporting effect size calculations to better inform science of biomarker discovery for frailty phenotypes. Fourth, molecule selections were often limited to a few nonspecific markers of inflammation. This reflects the state of science in biomarker development for frailty. Fifth, most of the included studies lacked control groups (i.e., non-cancer or healthy controls), thus it was challenging to determine the strength of association with frailty in the absence of solid tumors and treatments. In addition, IL-6, TNF-α, and CRP are repeatedly found to be elevated in a myriad of conditions linked to inflammation, such as obesity and smoking (45, 69). Therefore, future studies should include these relevant health characteristics as covariates in biomarker discovery studies. Additionally, there was heterogeneity in the type of treatments received among studies during treatment and/or post-treatment. Future studies may benefit from comparing the effects of different treatment types and modalities on frailty profiles and biomarker oscillation. Lastly, current literature lacks stratification by sex, race, and ethnicity, which decreases the generalizability and specificity of the results, and may also hinder our progress in developing targeted interventions.

## 5. Conclusion

In summary, IL-6, NLR, and GPS 2 emerged as potential biomarkers of frailty found in two or more of the included studies (Figure 3). Although IL-6 emerged as potential biomarker in five out of seven reports that measured this cytokine, findings remain inconsistent. Findings are inconclusive and were limited by number of reports found for all other measures. Our findings show that the current literature employs varying conceptual definitions of and instruments measuring frailty and that the genesis of frailty in solid tumors may be multifactorial, impacted by time since cancer diagnosis, treatments, and unique biology of individual solid tumors. Our findings highlight a need for further instrument validations and clear conceptual and operational definitions of frailty within the oncology field. Only two reports evaluated associations with biomarkers longitudinally. These two reports found that higher levels of inflammatory markers may serve as predictors of phenotypic frailty four weeks post-treatment (29) or at one year follow-up in patients with prostate cancer on ADT (26), however, further investigations are warranted with longer follow-up times. Post-treatment phenotypic frailty was captured four weeks (28, 29) and six months post-treatment (29), without data for pre-treatment (28) or during treatment (28, 29). The evidence highlights a substantial gap in long-term survivorship and frailty biomarkers evaluated longitudinally from pre-treatment to months and years post-treatment.

Collectively, the reports included in this review suggest that inflammatory pathways related to the proliferation of immune cells at the time of diagnosis and treatment are associated with frailty development and symptomology. Limited reports (one each) also implicate telomere shortening and epigenetic alterations such as perturbations in aging miRNAs as potential correlates of deficit accumulation frailty. Additionally, metabolic pathways underlying deficit accumulation frailty may be of potential value when identifying target biomarkers. Given the paucity of evidence across the diverse set of biomarkers searched, the field of frailty biomarkers in solid tumors is largely underexplored. Future studies will benefit from longitudinal studies with a comprehensive set of biomarkers adjusted for cancer stages, time since diagnosis and treatment, and type of treatment; larger sample sizes, robust control groups, and multiple time points by sex, gender, and race/ethnicity. Such investigations will aid the development of robust biomarker profiles, early identification of cancer survivors at risk for developing frailty, and timely referral to therapeutic interventions.

## Author contributions

DS and TA: conceptualization. DS, BP, DC, and TA: data curation and methodology. DS: formal analysis, project administration, and visualization. DS and BP: investigation and validation. TA: supervision. TA and DC: resources. DS, BP, VG, JG, and TA: writing—original draft. DS, BP, VG, DC, JG, and TA: writing—review and editing. All authors contributed to the article and approved the submitted version.

## Funding

This research was supported by the Intramural Research Program of the National Cancer Institute, National Institutes of Health.

## Conflict of interest

The authors declare that the research was conducted in the absence of any commercial or financial relationships that could be construed as a potential conflict of interest.

## Publisher’s note

All claims expressed in this article are solely those of the authors and do not necessarily represent those of their affiliated organizations, or those of the publisher, the editors and the reviewers. Any product that may be evaluated in this article, or claim that may be made by its manufacturer, is not guaranteed or endorsed by the publisher.
