# Supplementary material for: Blood-based biomarkers of frailty in solid tumors: a systematic review
Source: Front Public Health. 2023 May 4;11:1171243. doi: 10.3389/fpubh.2023.1171243 (PMC10193038; doi:10.3389/fpubh.2023.1171243)
Supplement: Supplementary file 1 [file data_sheet_1.pdf]

Supplementary Material

Blood-Based Biomarkers of Frailty in Solid Tumors: A Systematic Review

Dilorom Sass, Brennan Parmelee Streck, Vivian A. Guedes, Diane Cooper, Jennifer L. Guida, Terri S. Armstrong

*** Correspondence:**
Corresponding Author: Dilorom Sass, Ph.D. **Email:** dilorom.sass@gmail.com; dilijon08@gmail.com

Supplementary Table 1. Search Strategies for PubMed, Embase, and Web of Science

Database	Search strategy
**PubMed**	(“solid tumor”[tiab] OR “solid tumors”[tiab] OR “brain neoplasms”[Mesh] OR “brain neoplasms”[tiab] OR “Breast cancers”[tiab] OR “Prostatic neoplasms”[Mesh] OR “prostate cancers”[tiab] OR “Ovarian neoplasms”[Mesh] OR “Ovarian cancers”[tiab] OR “colonic neoplasms”[Mesh] OR “Colon cancers”[tiab] OR “Lung neoplasms”[Mesh] OR “Lung cancers”[tiab] OR “Pancreatic neoplasms”[Mesh] OR “Pancreatic cancers”[tiab]) AND (“Aging biomarkers”[tiab] OR Inflammation[Mesh] OR Inflammation[tiab] OR Neuroinflammation[tiab] OR “Extracellular vesicles”[Mesh] OR “Extracellular vesicles”[tiab] OR Exosomes[Mesh] OR exosomes[tiab] OR Cytokines[Mesh] OR cytokines[tiab] OR MicroRNAs[Mesh] OR miRNA[tiab] OR “DNA, mitochondrial”[Mesh] OR ”Mitochondrial DNA” [tiab] OR mtDNA[tiab] OR Telomere[Mesh] OR telomere[tiab] OR “telomere length”[tiab] OR “Frailty biomarkers”[tiab] OR “DNA methylation”[Mesh] OR “DNA methylation”[tiab] OR Inflamm-ageing[tiab] OR Inflammaging[tiab] OR P16INK4a[tiab] OR P19ARF[tiab] OR “Senescence-Associated Secretory Phenotype”[tiab] OR “31 Phosphocreatine recovery time”[tiab] OR “epigenetic clocks”[tiab] OR “epigenetic alterations”[tiab] OR “loss of proteostasis”[tiab] OR “Heat Shock Proteins”[tiab] OR “Deregulated Nutrient Sensing”[tiab] OR “Mitochondrial Dysfunction”[tiab]) AND (Aging[Mesh] OR aging[tiab] OR “Accelerated aging”[tiab] OR Frailty[Mesh] OR frailty[tiab] OR “Functional Decline”[tiab] OR “Deficit Accumulation”[tiab] OR “Decreased Physiologic Reserve”[tiab])<br><br>**Limits:** English; Human

**Embase**	(‘solid tumor’:ti,ab OR ‘solid tumors’:ti,ab OR ‘brain neoplasms’/exp/mj OR ‘brain neoplasms’:ti,ab OR ‘Breast cancers’:ti,ab OR ‘Prostatic neoplasms’/exp/mj OR ‘prostate cancers’:ti,ab OR ‘Ovarian neoplasms’/exp/mj OR ‘Ovarian cancers’:ti,ab OR ‘colonic neoplasms’/exp/mj OR ‘Colon cancers’:ti,ab OR ‘Lung neoplasms’/exp/mj OR ‘Lung cancers’:ti,ab OR ‘Pancreatic neoplasms’/exp/mj OR ‘Pancreatic cancers’:ti,ab) AND (‘Aging biomarkers’:ti,ab OR Inflammation/exp/mj OR Inflammation:ti,ab OR Neuroinflammation:ti,ab OR ‘Extracellular vesicles’/exp/mj OR ‘Extracellular vesicles’:ti,ab OR Exosomes/exp/mj OR exosomes:ti,ab OR Cytokines/exp/mj OR cytokines:ti,ab OR MicroRNAs/exp/mj OR miRNA:ti,ab OR ‘DNA, mitochondrial’/exp/mj OR ‘Mitochondrial DNA’ :ti,ab OR mtDNA:ti,ab OR Telomere/exp/mj OR telomere:ti,ab OR ‘telomere length’:ti,ab OR ‘Frailty biomarkers’:ti,ab OR ‘DNA methylation’/exp/mj OR ‘DNA methylation’:ti,ab OR Inflamm-ageing:ti,ab OR Inflammaging:ti,ab OR P16INK4a:ti,ab OR P19ARF:ti,ab OR ‘Senescence-Associated Secretory Phenotype’:ti,ab OR ‘31 Phosphocreatine recovery time’:ti,ab OR ‘epigenetic clocks’:ti,ab OR ‘epigenetic alterations’:ti,ab OR ‘loss of proteostasis’:ti,ab OR ‘Heat Shock Proteins’:ti,ab OR ‘Deregulated Nutrient Sensing’:ti,ab OR ‘Mitochondrial Dysfunction’:ti,ab) AND (Aging/exp/mj OR aging:ti,ab OR ‘Accelerated aging’:ti,ab OR Frailty/exp/mj OR frailty:ti,ab OR ‘Functional Decline’:ti,ab OR ‘Deficit Accumulation’:ti,ab OR ‘Decreased Physiologic Reserve’:ti,ab)
**Web of Science**	TS=(solid tumor OR solid tumors OR brain neoplasms OR brain neoplasms OR Breast cancers OR Prostatic neoplasms OR prostate cancers OR Ovarian neoplasms OR Ovarian cancers OR colonic neoplasms OR Colon cancers OR Lung neoplasms OR Lung cancers OR Pancreatic neoplasms OR Pancreatic cancers) AND TS=(Aging biomarkers OR Inflammation OR Inflammation OR Neuroinflammation OR Extracellular vesicles OR Extracellular vesicles OR Exosomes OR exosomes OR Cytokines OR cytokines OR MicroRNAs OR miRNA OR DNA, mitochondrial OR Mitochondrial DNA OR mtDNA OR Telomere OR telomere OR telomere length OR Frailty biomarkers OR DNA methylation OR DNA methylation OR Inflamm-ageing OR Inflammaging OR P16INK4a OR P19ARF OR Senescence-Associated Secretory Phenotype OR 31 Phosphocreatine recovery time OR epigenetic clocks OR epigenetic alterations OR loss of proteostasis OR Heat Shock Proteins OR Deregulated Nutrient Sensing OR Mitochondrial Dysfunction) AND TS=(Aging OR aging OR Accelerated aging OR Frailty OR frailty OR Functional Decline OR Deficit Accumulation OR Decreased Physiologic Reserve)

	**Limits:** English

Supplementary Table 2. Questions listed in the NHLBI quality assessment tools for the observational cohort and cross-sectional studies as well as case-control study.

**Questions - Quality assessment tool for observational cohort and cross-sectional studies (NHLBI, 2021a)**	**Questions - Quality assessment of case-control studies (NHLBI, 2021b)**
1.Was the research question or objective in this paper clearly stated?<br>2.Was the study population clearly specified and defined?<br>3.Was the participation rate of eligible persons at least 50%?<br>4.Were all the subjects selected or recruited from the same or similar populations (including the same time period)? Were inclusion and exclusion criteria for being in the study prespecified and applied uniformly to all participants?<br>5.Was a sample size justification, power description, or variance and effect estimates provided?<br>6. For the analyses in this paper, were the exposure(s) of interest measured prior to the outcome(s) being measured?<br>7.Was the timeframe sufficient so that one could reasonably expect to see an association between exposure and outcome if it existed?<br>8.For exposures that can vary in amount or level, did the study examine different levels of the exposure as related to the outcome (e.g., categories of exposure, or exposure measured as continuous variable)?<br>9.Were the exposure measures (independent variables) clearly defined, valid, reliable, and implemented consistently across all study participants?<br>10.Was the exposure(s) assessed more than once over time?<br>11.Were the outcome measures (dependent variables) clearly defined, valid, reliable, and	1.Was the research question or objective in this paper clearly stated and appropriate?<br>2.Was the study population clearly specified and defined?<br>3.Did the authors include a sample size justification?<br>4.Were controls selected or recruited from the same or similar population that gave rise to the cases (including the same timeframe)?<br>5.Were the definitions, inclusion and exclusion criteria, algorithms or processes used to identify or select cases and controls valid, reliable, and implemented consistently across all study participants?<br>6.Were the cases clearly defined and differentiated from controls?<br>7.If less than 100 percent of eligible cases and/or controls were selected for the study, were the cases and/or controls randomly selected from those eligible?<br>8.Was there use of concurrent controls?<br>9.Were the investigators able to confirm that the exposure/risk occurred prior to the development of the condition or event that defined a participant as a case?<br>10.Were the measures of exposure/risk clearly defined, valid, reliable, and implemented consistently (including the same time period) across all study participants?

implemented consistently across all study participants?<br>12. Were the outcome assessors blinded to the exposure status of participants?<br>13. Was loss to follow-up after baseline 20% or less?<br>14. Were key potential confounding variables measured and adjusted statistically for their impact on the relationship between exposure(s) and outcome(s)?	11. Were the assessors of exposure/risk blinded to the case or control status of participants?<br>12. Were key potential confounding variables measured and adjusted statistically in the analyses? If matching was used, did the investigators account for matching during study analysis?

Supplementary Table 3. Exclusion reasons of irrelevant reports based on titles and abstract screening (NHLBI, 2021a)

Exclusion categories for titles/abstracts	Number of reports excluded
Not in humans	87
Not in cancer or solid tumors	24
Not a primary research paper/ abstract/ review/ not research based (opinion paper)	93
Biomarkers of cancer progression/drug development	413
Did not measure biomarkers	19
Did not evaluate frailty/deficit accumulation	207

Supplementary Table 4. Reported Measures of Association (MOA)/Effect Sizes and P values for statistically significant results across 14 included studies.

**Author et al. year**	**MOA/Effect Size and P**
Brouwers et al. 2015	• **Balducci score**: IL-6 was higher in pre-frail and frail groups (MOA= NR, P = 0.019)<br>• **LOFS:** IL-6 also correlated with worse LOFS (r = -0.218, P = 0.013)
Buigues et al. 2020	• **≥ 6 months on ADT:** IL-6: (OR= 169.5; P = 0.013);<br>IL-8: (OR = 1.2; P = 0.014), lymphocyte count: (OR = 0.11; P = 0.036)<br>• **Follow up:** IL-6 associated with frailty: (OR = 96.8; P = 0.011)<br>• **Progression:** Higher baseline IL-6 (HR = 2.2; P < 0.05); and lower lymphocytes (HR = 1.2; P < 0.05) associated with frailty progression
Bylow et al. 2019	• Hemoglobin was lower in ADT group (MOA = NR; P < 0.01)
Corona et al. 2014	• MOA = NR for all the metabolites, age-adjusted results are at P < 0.05

Dalmasso et al. 2018	• Higher LOFS associated with miR-320b (ß = -0.623; P = 0.038), miR-374a (ß = 0.940; P = 0.0009)<br>• Lower miR-106b (ß= -1.145; P = 0.0171), miR-191 (ß= -1.461; P = 0.0054), miR-320b (ß= -0.776; P = 0.0266), miR-374a (ß= 1.209; P = 0.0026) served as predictors for total G8<br>• miR-301a (OR = 0.487; P = 0.0302) associated with higher frailty (fTRST)
Falandry et al. 2015	• GVS ≥ 3 associated with shorter TL group (OR = 2.06; P = 0.08)
Gilmore et al. 2020	• Greater pre-chemo cytokines associated with worse post-chemo frailty: IL-6 (MOA = NR; P =0.027); sTNFRI (MOA = NR; P = 0.039); sTNFRII (MOA = NR; P = 0.039).
Gilmore et al. 2021	• **Pre-chemo:** Total WBC (β = 0.039; P < 0.05), neutrophils (β = 0.039; P < 0.5), and NLR (β = 0.041; P < 0.05) were associated with frailty. **Post-chemo:** Total WBC (β = 0.021; P < 0.05), neutrophils (β = 0.24; P < 0.05), and NLR (β = 0.029; P < 0.01) were associated with frailty<br>• **Growth factors with chemotherapy pre- to post-chemo:** Total WBC (β = 0.023; P < 0.05), neutrophils (β = 0.023; P < 0.05), and NLR (β = 0.031; P < 0.01) were associated with frailty
Harneshaug et al. 2019	• GPS 2 was association with frailty (OR = 18.5, P = 0.006)
Hatse et al. 2014	• No significant differences found
Lealdini et al. 2015	• mGPS of 0 and frailty (MOA = NR; P = 0.021)<br>• mGPS of 2 and frailty (RR = 7.5; P = 0.012)
Navarro-Martinez et al. 2019	• **Cancer group:** higher IL-6 (OR = 56.9; P < 0.05) and fibrinogen (OR = 1.2; P < 0.05) associated with being frail<br>• **Control group:** CRP, IL-6, IL-8 and the severity of frailty syndrome (MOA = NR for all, P < 0.05)
Nishijima et al. 2017	• NLR was positively correlated with frailty (r = 0.220; P = 0.025) Patients in the top tertile of NLR (OR = 3.81; P = 0.031)
Ronning et al. 2010	• **FFP**: The frail group had significantly higher levels of CRP (MOA = NR, P = 0.025), IL-6 (MOA = NR; P < 0.025), TNF-α, where the pre-frail group vs. robust group (MOA = NR P < 0.025)<br>• **CGA:** CRP and IL-6 in the intermediate group vs. the fit group and in the frail group vs. the intermediate group (MOA = NR, P < 0.025). TNF-α levels were also significantly higher in frail vs intermediate group (MOA = NR, P < 0.025)

Abbreviations: CGA = Comprehensive Geriatric Assessment, CRP = C-reactive protein, chemo = chemotherapy, GPS = Glasgow Prognostic Score, FFP= Fried Frailty Phenotype, IL = interleukin, fTRST = Flemish Triage Risk Screening Tool, HR= hazard ratio, LOF = Leuven Oncogeriatric Frailty Score, miRNA = micro RNA, MOA= measure of association, mGPS = modified GPS, NLR = neutrophil to lymphocyte ratio, NR= not reported, TNF-α = tumor necrosis factor-α, sTNFR I = soluble TNF receptor I, OR= odds ratio, RR= relative ratio, WBC = white blood cells.

References:

NHLBI. (2021a). Study Quality Assessment Tools. *Quality assessment tool for observational cohort and cross-sectional studies.* Retrieved from https://www.nhlbi.nih.gov/health-topics/study-quality-assessment-tools

NHLBI. (2021b). Study Quality Assessment Tools. *Quality assessment of case-control studies*. Retrieved from https://www.nhlbi.nih.gov/health-topics/study-quality-assessment-tools
